# HIV-sensitive social protection for unemployed and out-of-school young women in Botswana: An exploratory study of barriers and solutions

**DOI:** 10.1371/journal.pone.0293824

**Published:** 2024-01-10

**Authors:** Ran van der Wal, Anne Cockcroft, Miriam Kobo, Leagajang Kgakole, Nobantu Marokaone, Mira Johri, Isabelle Vedel, Neil Andersson

**Affiliations:** 1 Department of Family Medicine, McGill University, Montreal, Québec, Canada; 2 CIET Trust, Gaborone, South-East, Botswana; 3 Centre de recherche du Centre Hospitalier de l’Université de Montréal (CRCHUM), Montréal, Québec, Canada; 4 Département de Gestion, d’évaluation, et de Politique de Santé, École de Santé Publique de l’Université de Montréal, Montréal, Québec, Canada; 5 Centro de Investigación de Enfermedades Tropicales, Universidad Autónoma de Guerrero, Acapulco, Guerrero, Mexico; University of Kwazulu-Natal, SOUTH AFRICA

## Abstract

Promotive social protection programs aim to increase income and capabilities and could help address structural drivers of HIV-vulnerability like poverty, lack of education and gender inequality. Unemployed and out-of-school young women bear the brunt of HIV infection in Botswana, but rarely benefit from such economic empowerment programs. Using a qualitative exploratory study design and a participatory research approach, we explored factors affecting perceived program benefit and potential solutions to barriers. Direct stakeholders (n = 146) included 87 unemployed and out-of-school young women and 59 program and technical officers in five intervention districts. Perceived barriers were identified in 20 semi-structured interviews (one intervention district) and 11 fuzzy cognitive maps. Co-constructed improvement recommendations were generated in deliberative dialogues. Analysis relied on Framework and the socioecological model. Overall, participants viewed existing programs in Botswana as ineffective and inadequate to empower vulnerable young women socially or economically. Factors affecting perceived program benefit related to programs, program officers, the young women, and their social and structural environment. Participants perceived barriers at every socioecological level. Young women’s lack of life and job skills, unhelpful attitudes, and irresponsible behaviors were personal-level barriers. At an interpersonal level, competing care responsibilities, lack of support from boyfriends and family, and negative peer influence impeded program benefit. Traditional venues for information dissemination, poverty, inequitable gender norms, and lack of coordination were community- and structural-level barriers. Improvement recommendations focused on improved outreach and peer approaches to implement potential solutions. Unemployed and out-of-school young women face multidimensional, interacting barriers that prevent benefit from available promotive social protection programs in Botswana. To become HIV-sensitive, these socioeconomic empowerment programs would need to accommodate or preferentially attract this key population. This requires more generous and comprehensive programs, a more client-centered program delivery, and improved coordination. Such structural changes require a holistic, intersectoral approach to HIV-sensitive social protection.

## 1. Introduction

The Government of Botswana has made massive investments to gain epidemiological control of HIV. It was the first African country to offer universal antiretroviral therapy in 2002 [1]. It eliminated mother-to-child transmission in 2021 [2] and approved pre-exposure prophylaxis for high risk groups, including for young women, in 2018 [3]. Botswana is one of few countries that achieved the 95-95-95 test-and-treat cascade to end AIDS as a public health threat by 2030: of all HIV-positive persons, 95.1% know their status, of which 98% are on HIV treatment, of which 97.9% achieved viral suppression [4]. Nonetheless, adult HIV prevalence is 20.8%, among the highest worldwide, with HIV prevalence in young women (15 to 30 years) up to three times as high as in young men [4].

Structural drivers of HIV-vulnerability like poverty, lack of education, and gender inequality undermine young women’s ability to act on HIV prevention choices [5]. Unemployed and out-of-school young women are especially vulnerable. Female unemployment in Sub-Saharan Africa predicts their disproportionate HIV burden [6]. Poverty increases young women’s economic dependence on men and constrains their ability to negotiate safe sex or leave unhealthy relationships [7]. Severe food insecurity, a proxy for extreme poverty, is associated with a twofold increased risk of recent HIV infection [8]. In Botswana, severe food insecurity is linked with HIV risk factors like unprotected sex, transactional sex, and early sexual debut [9]. In contrast, educational attainment is protective—each additional school year reduced HIV incidence by 8% [10]. Comparing schoolgirls and dropouts, school attendance in neighboring South Africa was associated with reduced HIV incidence; the difference in cumulative HIV incidence tripling in two years [11].

The Joint United Nations Programme on HIV and AIDS (UNAIDS) promoted HIV-sensitive social protection for its synergistic potential to achieve the Sustainable Development Goals (SDGs) of ending AIDS, poverty, and gender inequality by 2030 [12]. Social protection is defined as public or private transfers of resources to reduce socioeconomic vulnerability [13]. Social protection can be HIV-sensitive if it is inclusive of HIV-vulnerable groups at risk of, living with, or affected by, HIV-infection, and HIV-vulnerable groups are involved in its design and assessment [14–18]. Beyond protecting against, or preventing poverty, social protection can be *promotive*, in which case it aims to alleviate poverty by enhancing income and capabilities of the poor [19]. Commitment 6 of the UNAIDS *Fast-Track strategy to end AIDS by 2030* states that 75% of key populations, including adolescent girls and young women, should have benefited from HIV-sensitive social protection by 2020 [20]. In addition to traditional forms of social protection, it recommends socioeconomic, or promotive, approaches, including socioeconomic empowerment programs and second chance education [12].

A systematic review on HIV-sensitive social protection for unemployed and out-of-school young women in East and Southern Africa examined the effect of socioeconomic interventions on HIV and socioeconomic outcomes. The review found that livelihood training contributed positive socioeconomic outcomes but mixed HIV-related outcomes; microcredit contributed little to either outcome and was not recommended for this target group. Microgrants, savings, business and life skills contributed positive results for both outcome categories, and the review concluded such programs could be effective when comprehensive and adapted to target populations and local contexts [21].

With a mature and well-funded social protection system, taking 3.7% of the national GDP [22], Botswana might be well placed to achieve HIV-sensitive social protection. Beyond welfare and relief programs, Botswana offers several promotive social protection programs that might be leveraged against HIV-vulnerability. These include microloans, microenterprise development, productive asset transfers (small stock and poultry), agricultural inputs (seeds, fertilizer, or ploughing services), public works, apprenticeships and second chance education, and are offered by four different line ministries (Table 1). Although not designed for HIV prevention, these programs could be HIV-sensitive if they would be responsive to the needs and social situation of unemployed and out-of-school young women.

**Table 1:** Promotive social protection programs that enhance livelihood and capabilities in Botswana.

Income transfers through public works	Productive asset transfers	Income generation activity	Capabilities development
**Ipelegeng** *(Ministry of Local Government and Rural Development-MLGRD)* One-month rotating contracts (BWP567-US$54) without training. No requirements.	**LIMID** *(Ministry of Agriculture-MoA/MLGRD)* Productive asset transfers (small stock** and chickens) for destitute people*.	**Alternative Package Program** *(Social and Community Development (S&CD)-MLGRD)* Productive asset transfers or microenterprise support with microgrants, training, equipment for destitute people*.	**OSET** *(Ministry of Education and Skills Development-MOESD)* Free-of-charge primary education for illiterate adults
	**ISPAAD -horticulture** *(MoA)* Loans and inputs to support commercial farming. Requirements: business plan, land ownership or 10-year lease hold, access to water, and 40–60% upfront capital investment	**Youth Development Fund** *(YDF*, *Ministry of Youth*, *Sports and Culture-MYSC)* Mixed loans and grants (50%-50%) for microenterprise development with training. Requirements: business plan; unemployed and out-of-school youth 18–35 years	**BOCODOL** *(MOESD)* Distance learning for secondary education. School and examination fees waived for persons who did not receive Junior certificate (lower-level secondary education).
	**ISPAAD -farming** *(MoA)* Agricultural inputs: seeds, fertilizer, pesticides, loans, subsidies for ploughing and planting. Requirements: need to own, lease, or borrow fields.		**Tirelo Sechaba** *(YDF/MYSC)* Youth apprenticeships (20–30 years) in community service (health, education, agriculture) (BWP700-US$65).

*Destitute people: Individual income of BWP120 (US$11) or lower, or household incomes of BWP150 (US$14) or lower.

** the Government of Botswana uses the term small stock to refer to goats and sheep. APP: Alternative Package Programme; YDF: Youth Development Fund; ISPAAD: Integrated Support Programme for rain-fed Arable Agriculture Development; LIMID: Livestock Management and Infrastructure Development.

The Inter-ministerial National Structural Intervention Trial of HIV prevention (INSTRUCT), a multifaceted cluster randomized controlled trial (ISRCTN54878784) that addresses structural drivers of HIV vulnerability, aimed to leverage Botswana’s promotive social protection programs for HIV prevention [23]. Implemented by Botswana NGO Community Information, Empowerment and Transparency (CIET Trust) and the National AIDS Coordinating Agency, the INSTRUCT trial was implemented in five randomly selected intervention districts in Botswana. CIET Trust trained local young women to identify all unemployed and out-of-school young women in these districts through door-to-door visits and snowballing [24]. Trained peers conducted baseline interviews with unemployed and out-of-school young women (n = 3516). They showed video clips of available programs and invited them to 2-day information and empowerment workshops organized by CIET Trust. The CIET workshops offered life skills training and connected the young women with program officers who provided information about locally available programs [23,24].

The baseline interviews revealed that less than half of unemployed and out-of-school young women had applied to promotive social protection programs, and less than a third had been accepted. Excluding the easily accessible *Ipelegeng*, a public works program without any training, application and acceptance rates had been 33% and 10% respectively [24]. Against this background, we aim to explore why, according to direct stakeholders, promotive social protection programs in Botswana fail to benefit unemployed and out-of-school young women, and which potential solutions might remedy some of the barriers.

## 2. Methodology

Using a qualitative exploratory study design and a participatory research approach, we conducted semi-structured interviews and fuzzy cognitive maps to explore factors affecting perceived program benefit, and deliberative dialogue to identify stakeholder-designed improvement recommendations. We purposively sampled individuals with a direct stake in, experience with, or knowledge of, promotive social protection programs. The two stakeholder groups consisted of young women, defined as unemployed and out-of-school, aged 18 to 30 years, and officers: program officers who deliver socioeconomic programs and technical officers from land and water boards, as land and water were prerequisites for several programs. To take contextual differences into account, we organized mapping and dialogue workshops in all five intervention districts. Due to budget and time constraints, semi-structured interviews occurred only in Moshupa sub-district (Table 2).

**Table 2:** Data collection methods and study participants.

Data collection	Events	Young women	Officers	Selection criteria
Fuzzy cognitive mapping workshops	11	40	26	Separate groups of young women who had participated in 2015–2017 CIET Trust information and empowerment workshops and local program and technical officers in five intervention districts. In Moshupa sub-district, young women had also participated in semi-structured interviews.
Deliberative dialogue workshops	5	31	29	Combined groups of young women and officers who participated in FCM workshops in five intervention districts.
Semi-structured interviews	20	16	4	Young women who had participated in 2015–2017 CIET Trust information and empowerment workshops and one program officer per available program in Moshupa sub-district.
**Total**	**36**	**87**	**59**	**Total participants: 146**

YW: Unemployed and out-of-school young woman; Officers: Program and technical officers; CIET Trust: Botswana research NGO.

### 2.1 Study setting

Botswana is a Southern African country roughly the size of France with a population of 2.3 million inhabitants [25]. It is prone to droughts due to its semi-arid climate, extreme temperatures, and uneven rainfall. Two thirds of the country is covered in infertile soils but arable farming is feasible in the eastern parts of Botswana [26]. Despite its upper middle-income country status, nearly 60% of Batswana lived in poverty in 2021, and 14.1% in extreme poverty [27]. The unemployment rate is 24.5% overall, 32.2% for youth, and much higher in rural than in urban areas [28]. Of the 37.5% of youth that is both unemployed and out-of-school, 57.7% is female [28].

Three of the five randomly selected INSTRUCT intervention districts, including Moshupa sub-district, are situated within a 1–1.5-hour drive from Gaborone, Botswana’s capital, in the most populous south-eastern part of the country that borders South Africa. A fourth intervention district borders Namibia in the west and is part of the Kalahari Desert. It is sparsely populated by a minority tribe, and suffers among the highest poverty and unemployment levels in the country [28,29]. The fifth intervention district is part of Central District and borders Zimbabwe. With a relatively mild climate, it is the most agriculturally productive district in Botswana [30].

### 2.2 Data collection and analysis

#### 2.2.1 Fuzzy cognitive mapping and deliberative dialogue

We used a two-stage participatory process involving fuzzy cognitive mapping (FCM) to identify factors impeding program benefit and deliberative dialogue to explore improvement options. Participatory research involves co-creation of knowledge with those affected by the study, to affect social change through an empowering process of social learning [31]. It offers the framework to place local evidence, lived experience, and innovation by participants at the center [32].

FCM combines cognitive maps with fuzzy logic and uses local explanations for analysis [33]. It therefore allows integration of non-traditional expert knowledge in planning and decision-making processes [34]. FCM is intuitive, culturally fairly neutral, and appropriate for research with marginalized populations, as their maps can be placed on par with, or even outweigh, maps produced by more advantaged groups [35].

In each of the five intervention districts, we first organized FCM workshops with separate stakeholder groups of young women and officers, followed by a deliberative dialogue workshop, which combined the two groups. Officers were purposively sampled from each of the programs and land and water boards present in each district. We purposively selected young women from the population of unemployed and out-of-school young women who had participated in information and empowerment workshops CIET Trust in 2015–2017 [23,24] on the basis of willingness to participate in participatory workshops, whilst ensuring representation based on rural and urban location. To keep workshops manageable and meaningful, we limited the group size to a maximum of 10 participants for FCM and 16 for deliberative dialogues. In Moshupa sub-district, we selected young women who had participated in semi-structured interviews and organized two FCM workshops, as their number exceeded the maximum group size. Overall, 11 FCM and 5 deliberative dialogue workshops took place throughout 2017 in all five intervention districts, each lasting around three hours (Table 2).

Setswana-speaking CIET staff facilitated the participatory workshops and took written notes. FCM workshops started with facilitators writing down the outcome of “young women not benefitting from programs” in the center of a large whiteboard. Participants wrote all underlying reasons they could think of on individual cards. After comparing cards and removing duplicates, they clustered cards with similar concepts. They placed the cards on the whiteboard and drew circles around each concept. Participants subsequently linked these concepts with arrows to represent perceived causal pathways. The “fuzzifying” involved assigning weights to each causal link to indicate its perceived relative importance. Weights ranged from one to five, from least to most important.

In subsequent deliberative dialogue workshops, the two stakeholder groups collaboratively analyzed the maps made by each group (see analysis section) [33]. Together, they identified two or three priority problems, proposed context-sensitive solutions, and considered the feasibility of proposed solutions [33]. While deliberative dialogue focuses on problem solving, the process also supports the development of critical consciousness among different social groups by careful consideration of pros and cons of proposed solutions [36]. The engagement of both service users and providers could therefore contribute feasible, acceptable and context-sensitive solutions whilst strengthening feelings of ownership and support for their implementation [37].

#### 2.2.2 Semi-structured interviews

In May and June 2017, a local woman interviewer conducted semi-structured interviews with young women in Setswana in Moshupa sub-district, a mainly rural district, close to the national capital, Gaborone. We purposively sampled 16 young women from a pool of unemployed and out-of-school young women who had attended CIET Trust information and empowerment workshops in Moshupa sub-district in 2015–2017. Maximum variation sampling criteria [38] included program application (Y/N), application status, location, and type of program. The interview guide for young women covered their experiences with applications and program officer support (S3 Appendix). The lead author (RW) conducted semi-structured interviews with four program officers who were fluent in English, one from each ministry implementing socioeconomic programs in Moshupa sub-district. The guide for program officers covered their views of, and experiences with, programs, program delivery, and accessibility for young women (S3 Appendix). Interviewers wrote down responses and took observational notes. Interviews lasted 45 to 90 minutes and took place at locations of participants’ choosing, mostly their homes or offices. Anonymized data extractions grouped per interview question can be found in S3 Appendix.

#### 2.2.3 Analysis

Thematic analysis relied on Framework, a matrix-based method developed for applied qualitative research [39]. We used an inductive-deductive sequential approach to analysis. Starting from interview questions, RW systematically coded interview data into themes and main categories. For the FCM, participants compared cognitive maps with pattern matching tables (23) to identify convergent concepts, and to prioritize issues for deliberative dialogue. Subsequently, a group of young women and facilitators condensed FCM concepts into themes [40]. Last, RW thematically assigned FCM themes to the main categories that resulted from the analysis of interviews (S1 Appendix). For the deliberative dialogue, RW used thematic analysis and coded themes into the same main categories (S2 Appendix). For the overall analysis of findings derived from the three different methods, RW integrated findings in a Framework matrix, juxtaposing methods and participant groups with main categories. This supported methodological triangulation and analysis within and across main categories and participant groups [39]. Only authors involved in direct data collection had access to non-anonymized data during or after data collection.

To emphasize that interacting barriers influence program benefit at multiple levels, RW grouped all findings into pre-determined conceptual groups of personal, interpersonal, community, and structural level barriers according to the socioecological framework of Bronfenbrenner (1977) and McLeroy et al (1988) [41,42]. At personal level, the framework focuses on individual characteristics like knowledge, skills, attitudes, and behaviors that may facilitate or impede program benefit. The interpersonal level looks at family and other direct social dynamics, whereas the community level focuses on the social and physical environment of the greater community. Finally, at structural level barriers are found in the wider sociocultural, economic, legal, political, and organizational environment of Botswana in which promotive social protection programs are offered.

### 2.3 Ethical approval

Botswana’s Health Research and Development Committee (HRDC00724) and McGill University (A12-B72-18A) granted ethical approval. Reading from a prepared script (S4 Appendix), we informed potential participants of the research purpose and rights of participants. We stressed the confidentiality of their responses, the voluntary nature of participation, and the right to not answer questions or stop participating at any time. We obtained oral informed consent from participants prior to interviews and workshops, as is accepted in Botswana [43,44].

## 3. Findings

### 3.1 Overview

We conducted 11 FCM workshops and five deliberative dialogue workshops with 66 and 60 participants respectively across five intervention districts in Botswana. The FCM generated 168 concepts, of which 37 appeared on maps of both young women and officers in at least three districts (S1 Appendix). Most concepts (n = 115) appeared on both stakeholder maps in at least one district, but the pattern matching table showed them to be more frequent in one or the other group (S2 Appendix). For example, only one officer map identified bad or improper officer behavior whereas this featured on all young women’s maps. Eleven concepts were solely mentioned by young women and five solely by officers.

We conducted 20 semi-structured interviews in Moshupa sub-district (Table 2), including four with program officers delivering locally available promotive social protection programs. Table 3 shows the demographic characteristics of the four program officers and 16 young women and the programs they had (successfully) applied to.

**Table 3:** Demographic characteristics interview participants & associated programs.

#	Age (y.)	Sex	Marital status & #children	P*lace	(Application to) promotive social protection programs available in Moshupa sub-district	Application information & decision time
PO						
1	33	F		Moshupa*	APP (microenterprise packages for destitute persons)	Few YW apply
2	34	F		Moshupa*	YDF (microenterprise development, Tirelo Sechaba)	Few YW apply
3	55	M		Moshupa*	ISPAAD (agricultural inputs and horticulture)	Few YW apply
4	33	F		Moshupa*	LIMID (chickens/small stock for destitute persons)	Many YW apply
YW						
1	24	F	Single, 0	Moshupa*	LIMID small stock	accepted
2	25	F	Single, 0	Moshupa*	LIMID small stock	refused
					Tirelo Sechaba	accepted
3	26	F	Single, 1	Moshupa*	LIMID, APP (catering)	refused
4	21	F	Single, 0	Moshupa*	Did not apply to any program	-
5	22	F	Single, 1	Kgomokasitwa	LIMID small stock	refused
					APP (catering)	accepted
6	25	F	Single, 1	Kgomokasitwa	LIMID small stock	refused
					APP (chickens)	accepted
					APP (catering)	pending, 2 y.
7	22	F	Single, 0	Manyana	LIMID small stock	accepted, waiting for disbursement 2 y.
8	23	F	Single, 0	Manyana	LIMID small stock	pending, 2 y.
					BOCODOL	withdrawal
9	21	F	Single, 1	Manyana	APP (chickens)	pending, 1 y.
10	21	F	Single, 1	Tswaane	Did not apply to any program	-
11	24	F	Single, 2	Tswaane	Tirelo Sechaba	accepted
12	25	F	Single, 2	Ranaka	Did not apply to any program	-
13	24	F	Single, 1	Pitseng	APP (tent hire)	pending, 2 y.
14	21	F	Single, 1	Pitseng	Ipelegeng	accepted
15	20	F	Single, 2	Pitseng	APP (tent hire)	pending
16	29	F	Single, 1	Sesung	APP (chickens)	refused

#: Number; PO: Program officer; YW: Unemployed and out-of-school young woman; y.: Years; F: Female, M: Male

*urban location; APP: Alternative Package Programme; YDF: Youth Development Fund; ISPAAD: Integrated Support Programme for rain-fed Arable Agriculture Development; LIMID: Livestock Management and Infrastructure Development; Tirelo Sechaba: Youth apprenticeships; destitute: Individual income of BWP120 (US$11) or lower, or household incomes of BWP150 (US$14) or lower.

### 3.2. Factors affecting program benefit

We first report findings according to the main five Framework categories of program, program officer, young women, social, and structural factors that affect program benefit for unemployed and out-of-school young women. The Framework matrix (Table 4) shows findings per method and participant group juxtaposed with main categories. For the matrix, FCM themes we selected to report had been mentioned by at least 50% of the maps. We then summarize the barriers to perceived program benefit with the socioecological model that categorizes barriers at personal-, interpersonal-, community- and structural-level barriers.

**Table 4:** Framework overview of main factors affecting program benefit per method and participant group.

Methods & participants	Program factors	Program officer factors	Young women factors	Social factors	Structural factors
Interviews Young women	• Application process is easy • Decisions take too long • Rural areas require more attention • Need for better outreach	• Half of YW found POs helpful • A third found POs unhelpful • Frustration with the process transformed into anger with POs	• Despair in YW expressed as resentment and hopelessness • Patience and determination • Few YW with positive feelings (happiness, pride, satisfaction)	• Most YW knew someone assisted by the program • Group solidarity; learning from peers • Mobilization of YW • Competing childcare responsibilities	• (Extreme) poverty • Need to support family (bread winners) • Community-level challenges
Interviews Program officers	• Easy application process (most programs) • Some programs are not accessible for YW • Most programs are not sustainable	• Effort to support YW • Feelings of demotivation • Upstream barriers	• YW lack technical and social skills • Negative attitudes among YW • YW’s preferences and expectations do not match programs on offer • Few YW in programs	• YW lack a supportive social environment	• YW lack access to land and water • Lack of coordination between programs or with land/water boards • Generalized poverty prevents program success
FCM YW	• Poor outreach • Process is complicated/too long (some programs) • Difficulty of (physical) access in remote areas: lack of transport* and POs not visiting remote areas*	• Bad/improper PO behavior • POs unhelpful/unfriendly • POs are unfair*	• YW lack knowledge/skills • YW lack self-confidence • Unhelpful attitudes and behaviors among YW • YW hold negative views of programs	• Competing household and childcare responsibilities • Unsupportive partners • Jealousy and competition between YW* • Stigma associated with poverty programs*	• Poverty • Institutional barriers (lack of access to land and water)
FCM Officers	• Poor outreach • Process is complicated/too long (some programs) Programs are unsuitable, including Ipelegeng**		• YW lack knowledge/skills • YW lack self-confidence • Unhelpful attitudes and behavior among YW • YW hold negative views of programs	• Competing household and childcare responsibilities	• Poverty • Social norms • Institutional barriers (lack of access to land/water; lack of coordination**; policies** and legislation**)
DD improvement recommen-dations	• Diversify venues for outreach: clinics, churches, school kids passing on message to YW, social media, market days • Involve YW in outreach	• POs help fill out forms • POs improve efficiency, client friendliness • Performance indicators for POs to bring YW onto programs	• Advertise options for free return to secondary education • Train YW to train peers to fill out complicated applications • YW could form groups to approach POs for vocational training	• Involve community and boyfriends in outreach • Leverage role models for YW • Form YW support groups	• Improve coordination between programs and with land/water boards • Involve national levels for support

Main themes reported per data source (horizontal) or by factor (vertical). FCM themes in the table were reported by at least 50% of stakeholder groups.

*FCM concepts only mentioned by young women

**only mentioned by officers. Abbreviations: YW: unemployed and out-of-school young women; PO: Program officers; FCM: Fuzzy cognitive maps; DD: Deliberative dialogue.

#### 3.2.1. Program factors

Poor program information dissemination, long and complicated application processes, and unsuitable programs were the main program barriers. Most program information dissemination occurs during Kgotla meetings, which are public community meetings presided over by the chief and locally elected officers and mostly attended by older persons. Feeling uncomfortable and judged, few young women attended Kgotla meetings, hence would not learn about available economic empowerment programs or calls for applications.

Application to poverty eradication programs was mostly considered easy, merely involving registration with an identity card. Application to YDF microenterprise and ISPAAD horticulture programs were too complicated and financially inaccessible, due to the detailed business plans they required, and the 40–60% upfront co-investment (ISPAAD horticulture).

The lengthy process was a major problem. Applicants waited several years to receive an initial decision, and hardly received any feedback. Once accepted, they experienced long delays in disbursement of funds or materials. Although many took the slow process as a lesson in patience, it profoundly disheartened others.

“*First*, *I was happy and thought it would not take much time but later I became disappointed*, *lost morale*. *It looks like government programs were meant to hurt our feelings*. *This made me lose interest in applying for any government program*.*” (YW8)*

Program officers explained that upstream barriers contributed to processing delays. They also suffered long delays and uncertainty about when and how much funding would be disbursed. They agreed regular feedback to young women could assuage feelings of frustration. With programs understaffed and underfunded they lacked time, money, and material resources to support beneficiaries.

“*It takes a long time before we get the funds*. *We still have a one-year backlog because we do not receive enough funds*. *We keep registering them if they’re eligible but maybe we should know how much we receive and stop registration when we reach the limit*.*” (PO4)*

Officers perceived many programs as unsuitable and unsustainable. Microenterprise projects developed through the Alternative Package Program hardly generated a living wage and the more ambitious microenterprises supported by the Youth Development Fund often failed, in part due to incompatibility with local demand and conditions.

“*They are not sustainable*. *Poverty eradication programs don’t get them out of poverty… I emphasize education because projects are short-term*. *They start a tuck shop; then a supermarket opens*, *and nobody comes*.*” (PO1)*

Some young women suggested programs should have a special focus on young women, either through specialized offices or through differential processes with improved counselling and follow up. Young women were grateful to generate even a small income, sometimes benefitting from demand generated by the government itself.

“*It is not much*, *but at the end of the month I am able to survive… I can make between 900–1000 (US$85)*. *This includes chips and Russians (hotdog sausages)*. *At least for (government) tenders I make P2500 (US$225)*.*” (YW5)*

Although young women seemed to appreciate *Tirelo Sechaba*, YDF’s work-integrated learning program, the lack of apprenticeship positions, especially in rural areas, made opportunities for work-integrated learning rare. Two young women with *Tirelo Sechaba* positions counted themselves lucky, but as breadwinners, the monthly allowance (US$60) was insufficient to feed their families. The program officer stressed *Tirelo Sechabo* allowances were stipends and that some youth were subsequently hired for permanent employment.

An *Ipelegeng* beneficiary considered the public works program as easy money that brought food to the table. Program officers viewed *Ipelegeng* rather as a barrier. Without any training component and very low allowances (US$54 per month), they felt it prevented participants from pursuing more sustainable opportunities while keeping them in poverty.

#### 3.2.2. Program officer factors

Cognitive maps showed bad program officer behavior and attitudes, which ranged from extortion and exploitation to contempt and officers being unhelpful and unfriendly. Young women perceived officers as unfair accusing them of tribalism, favoritism, and discrimination against the poor. Some officers even requested bribes and sexual favors.

In interviews, most of the young women recounted positive personal experiences with program officers, who they perceived as approachable and helpful, with some going the extra mile of signing up young women during home visits. The lengthy process and lack of follow-up or application success changed these positive feelings into feelings of betrayal and disappointment.

“*The program officer was humble*, *made sure I understood what was discussed*. *First*, *I felt very happy because the whole process came to my doorstep*, *but I started to lose hope and now I don’t trust government officers*. *First it was like the best program*, *I become emotional every time I think about it*. *It’s like I’m a failure*.*” (YW9)*

Program officers reported putting a lot of effort into engaging young women. They felt they went above and beyond routine outreach efforts to motivate and support their clients, even using personal resources. Program officers were aware of accusations of corruption against them but explained clients did not realize they faced upstream barriers. The lack of supervisory, financial, and material support, notably the lack of vehicles, computers, and airtime, impeded their capacity to offer quality services. Unconvinced of the benefits or sustainability of programs, some program officers preferred orienting youth towards further education rather than to self-employment. The combined burden of upstream barriers, an uninterested clientele, and lack of success stories contributed to feelings of demotivation and hopelessness among program officers.

“*We do orientations; we also do one-on-one*…*explain what we do*. *Workshops for business awareness creation*, *to open their minds*…*sometimes they’re just not interested at all*.*” (PO2)*

“*We are philanthropists here*. *Our phone can’t access cell phones*, *so to alert (young women) about adverts*, *I use my own bundle to call*. *Most of the time we use our own resources*, *own car*, *own petrol…I feel demotivated*, *I have been in this job for 10 years but did not see any success*.*” (PO1)*

#### 3.2.3. Young women factors

Perceived lack of technical and social skills, and unhelpful attitudes and behaviors impeded young women to benefit from programs. Both stakeholder groups mentioned lack of formal education, basic literacy skills and lack of vocational or business skills.

“*Many don’t have skills*. *Maybe that’s why they’re reluctant to apply*. *For example*, *she says she wants to do a hair salon*, *but she doesn’t know anything about hair*. *They have to produce a certificate and prove they have skills*.*” (PO2)*

A profound lack of life skills, including the lack of self-confidence, self-esteem, assertiveness, and communication skills prevented young women from receiving program information, applying to programs, or benefitting from them. Young women were too shy to proactively seek program information. They felt intimidated by some programs, perceiving them as too big and too time consuming, but admitted they lacked commitment or interest in entrepreneurial programs. Participants perceived young women as irresponsible, impatient, disrespectful, lazy, with unrealistic expectations.

Officers believed young women preferred white collar jobs to farming in rural areas, even if these paid less. They felt young women expected quick cash and government involvement in running the enterprise and bringing products to market. They were exasperated with the perceived “short-sightedness” and “spoilt character” of young women and wondered whether life skills and psychosocial support would not benefit them more than economic empowerment programs.

“*We give them training*. *We visit them weekly*. *We don’t know what to do*. *Maybe S&CD should give psychosocial support for encouragement and life skills*.*” (PO3)*

Dominant feelings among the young women we interviewed were dejection and despair. They were frustrated with the lengthy process, resented being rejected, and felt hopeless with the lack of prospects to escape poverty. Several repeated young women-blaming narratives that they should not depend on boyfriends or parents, and avoid drugs, alcohol, and teenage pregnancy.

“*The programs can improve our lives*. *They can keep us busy form being engaged in alcohol*, *teenage pregnancies and can create employment*.*” (YW6)*

The few young women who expressed positive feelings like happiness, pride or satisfaction had enjoyed recent application success. Nonetheless, many young women stressed their positive attributes like patience, perseverance, initiative, and solidarity. Several young women took the possibility of self-employment very seriously, reflected in their ideas to turn potential assets into sustainable businesses.

“*(I would) sell to junior secondary school*. *I know there’s a shortage of egg supply in the village…shops purchase their eggs from Gaborone*. *The manure from the poultry would be supplied to commercial gardens owners*.*” (YW9)*

#### 3.2.4. Social factors

Social factors included care responsibilities, lack of support, and peer influence. Competing household or childcare responsibilities prevented young women from returning to school or engaging in economic activity. Program officers pointed to negative family influence contributing to lethargy and intergenerational poverty, but many young women reported having positive role models who motivated them to apply. Some family members helped with childcare so young women could work, but others felt their family constrained their choices.

“*I wanted to apply…but have to stay with two children…and a…baby*, *so this seems to have shuttered my dreams*. *Maybe*, *when my mother returns…rather than running away and leaving them with me*.*” (YW10)*

Beyond lack of family support, other social barriers pertained to boyfriends and community members. Unsupportive boyfriends were a major barrier, as some refused to let their girlfriends apply. At community-level, wealthier community members were hesitant to get involved in young women’s economic activities, denying them a place of operation, or selling small stock to them.

“*I was discouraged by a farmer refusing to offer quotations for goats*, *saying it takes time for the government to pay them after supplying the small stock*.*” (YW2)*

Some officers perceived socioeconomic programs as contributors to the decline of traditional values like community spirit and reciprocity, as youth were now unwilling to work for someone else.

“*I think*…*(programs)…destroyed the neighborliness of Botswana…We have a shortage of (agricultural) labor here*. *They have that pride whereby they cannot work for someone else*, *even if it is a relative*.*” (PO3)*

Peer influence seemed to have both negative and positive effects. Seeing peers rejected, hearing about bad experiences, or the stigma of being in a poverty program discouraged young women from applying to programs. Envy and jealousy contributed to an environment whereby young women competed rather than collaborated, withholding relevant information from each other. Yet, young women also advocated for voice and solidarity. Several interviewed young women mentioned wanting to learn from successful peers. Some wanted to mobilize peers to improve their destiny, to hold program officers accountable, or to denounce them.

“*We should be creative*, *come up with initiatives aimed at employment creation*. *We should interact to learn business ideas and skills*, *share ideas*, *experiences*, *meet successful youths*. *We need to work as a team; this will motivate us*.*” (YW3)*

#### 3.2.5. Structural factors

Poverty, social norms, gender inequity, and institutional barriers were structural barriers. Poverty prevented young women from applying to programs if this involved transportation costs, or accumulating capital to start and support entrepreneurial projects. Poverty could be a barrier to ongoing education, as some young women reported that schools withheld diplomas when school fees were left unpaid.

“*I was admitted (to vocational education) but not able to attend because I did not have the certificate as I had been unable to pay school fees*… *No one is working in my family*, *so life is not easy*.*” (YW15)*

According to some program officers, the context of generalized unemployment, where even highly educated young women had to resort to transactional sex, made it nearly impossible for uneducated young women to thrive in socioeconomic programs.

“*Even when they do tertiary education*, *they don’t find work*. *They get into relationships for money*. *Then they get kids*. *It is frustrating to have studied four years in Gaborone and then you sit at home in Moshupa*.*” (PO1)*

Despite available jobs in farming, participants viewed agricultural jobs as unsuitable for young women, with few female role models successfully engaged in farming. Poultry and small stock programs offered by LIMID, APP, and YDF, were relatively easy to obtain, but provided only a small number of goats. Breeding them into larger herds took too long, resulting in the poorest families consuming them. Lack of money hampered prevention of zoonotic diseases. A young woman reported losing her entire flock of Tswana chickens to Newcastle disease. Having enjoyed some prosperity, her subsequent downfall left her in even greater despair.

“I *experienced a natural disaster…lost all chickens…I reported to program officers…but I was only told that funds were finished*. *I used to help my family…buying them food and school uniforms*. *I later felt I am a more needy person now*.*” (YW6)*

Institutional barriers included lack of coordination and lack of access to land and water. Officers reported a lack of coordination between programs, but also between programs and land and water boards. They perceived program duplication, inefficiencies, and competition due to the lack of monitoring and evaluation and lack of coordination between different ministries.

“*We offer the same program*, *but YDF pays more for goats than us*.*” (PO4)*

“*Programs are not being evaluated for effectiveness…There is S&CD*, *LIMID*, *Youth -we offer the same thing*. *Why can’t these programs be centralized*? *There is no linkage*. *A person could benefit from the same thing at different departments*.*” (PO1)*

Lack of access to land, water, or a place of operation made young women ineligible for programs. To receive small stock, they required access to water; for farming, access to land. Few young women owned or applied for land or had proof they could borrow land. Lack of coordination between programs and land and water boards sometimes resulted in having to postpone receiving assistance while waiting for land or water allocation. Land laws excluding young women, and the lack of political will to address it, were other institutional barriers on officer maps.

“*You need to have land*. *It may take a long time that a land allocation request will be approved*. *…They need to have a sustainable*, *continuous and productive water source*, *for example a borehole or river*, *for the entire life of the project*.*” (PO3)*

### 3.3. The socioecological model: Barriers to program benefit

**Fig 1:**
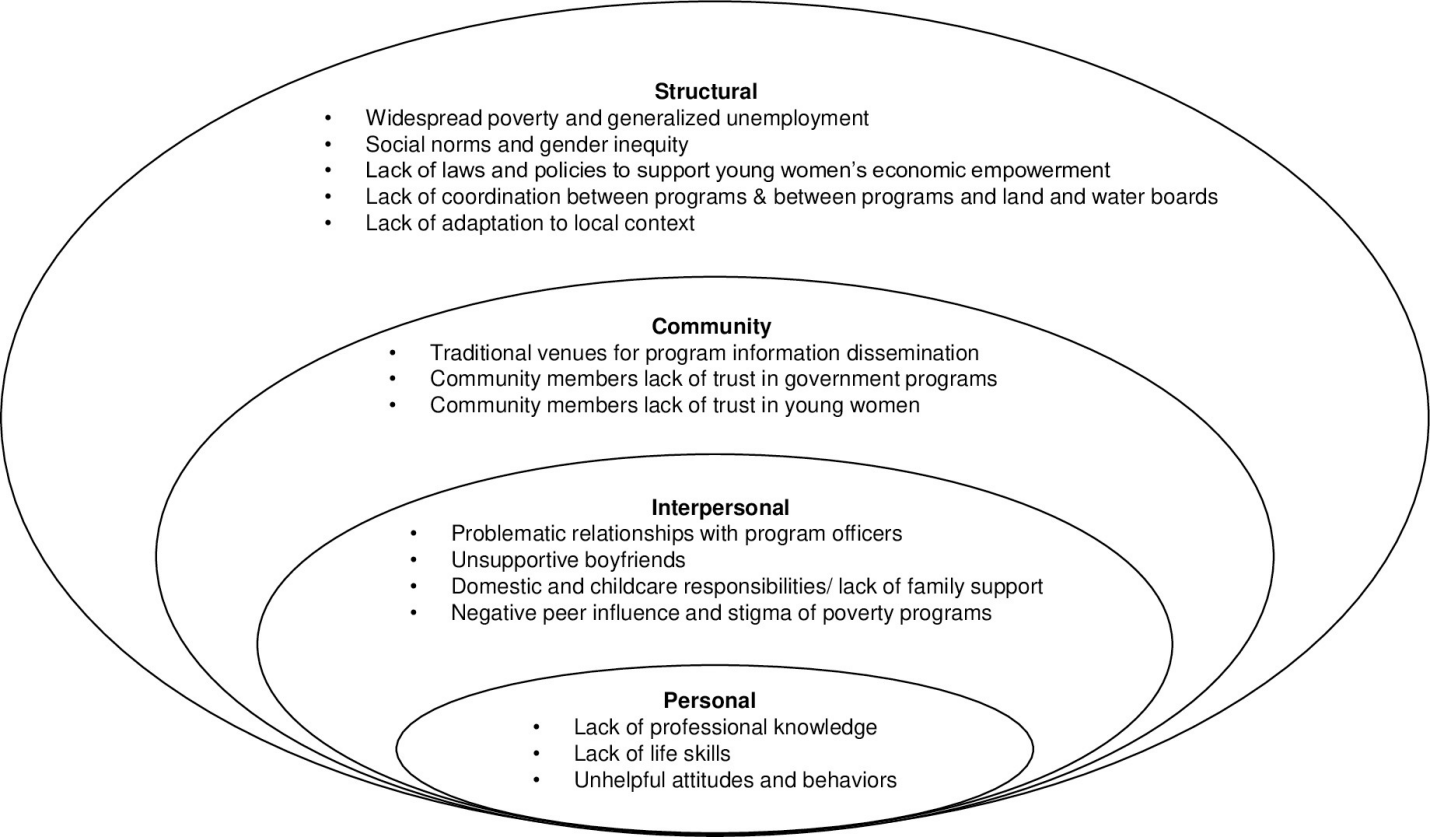
Socioecological model of barriers that impede benefit from promotive social protection programs by unemployed and out-of-school young women in Botswana.

Participants perceived multifaceted and interacting barriers at every socioecological level. Fig 1. summarizes our findings depicting how barriers at the personal, interpersonal, community, and structural levels constrained unemployed and out-of-school young women to benefit from promotive social protection programs in Botswana.

### 3.4. Stakeholder-designed improvement recommendations

Improving outreach through diversification of venues and means of communication was the main recommendation theme emerging from the deliberative dialogue workshops (S2 Appendix). Besides traditional venues like the *kgotla* and village development committees, program information could be spread on market days, through youth committees, churches, social media, radio, or through children who could receive information at school and pass it on. Information could reach young women attending child welfare sessions at local clinics. To reach the most rural populations, nurses could disseminate program information through mobile clinics in the *masimo* (agricultural lands outside villages). Distance learning opportunities for primary and secondary education (OSEC and BOCODOL) could ameliorate young women’s lack of education. These second chance education programs were free of charge for young women who had dropped out prior to Form 3, but this was not widely known, and participants felt it should be better advertised.

Another prominent theme was active engagement of young women in the implementation of potential solutions. Young women could collate and disseminate program information, help fill out complicated applications, receive training to train peers, or form groups to proactively request vocational training. Some suggested embedding such groups in customary administrative systems like *kgotlana* (village wards). Some program officers proposed funding such groups.

Improvement recommendations for program officers mostly involved hard-to-measure solutions like improving efficiency, client friendliness and assistance. One group proposed including performance targets for program officers to bring unemployed and out-of-school young women onto programs.

To overcome social barriers, suggestions involved leveraging positive role models and involving community and boyfriends in outreach and training. Opportunities for structural improvements pertained to land and water access. Participants suggested programs collaborate and proposed establishing formal agreements between programs, and between programs and land and water boards, while leveraging hierarchy through district-level coordination by the district commissioner. They admitted this would require policy action at national level as well.

## 4. Discussion

This study explored why promotive social protection programs offered by the government of Botswana fail to benefit unemployed and out-of-school young women. We investigated perceived barriers and potential solutions in the context of HIV-sensitive social protection, as these programs could reduce HIV vulnerability by reducing social and economic disadvantage.

Many study participants perceived current programs as ineffective and inadequate to empower young women socially or economically, reporting barriers at every socioecological level [41,42]. The lack of life skills at a personal level is of crucial importance for HIV-sensitive social protection, as life skills do not only increase socioeconomic success among unemployed and out-of-school young women [45,46], but also help improve sexual negotiation and reduce sexual risk behaviors [47,48]. Complementing available programs with life skills training might facilitate social interactions, including with program officers. Professional skills like business and financial skills could help young women plan, save, and spend responsibly and improve their self-efficacy, a life skill also associated with reduced sexual risk behavior [49–51]. Some scholars suggest that *Ipelegeng*, the most easily accessible program for young women, could be made more HIV-sensitive with a skills development component and active linking to other socioeconomic programs [52]. In addition, given young women’s negative attitudes and behaviors, mentoring of job and life skills might benefit young women transition into new livelihoods [53]; dialogue groups suggested peer mentoring programs would be welcomed.

At an interpersonal level, negative peer influence was expressed in reported jealousy and competition with peers. It seems plausible that poverty might force young women to compete for the few available resources. The stigma of being in a poverty program may be another expression of negative peer influence. Peer pressure and “symbol capital” among youth are long-recognized phenomena in the literature on transactional sex [54]. Transactional sex is commonly accepted to acquire nice hair, fashionable clothes, cell phones and other symbols of a successful life [55]. Associations with poverty programs would undermine the social status young women might want to construct for themselves.

Programs seem to take little account of the needs and social situation of unemployed and out-of-school young women. Many young women are single mothers suffering time poverty due to domestic and childcare responsibilities. Offering childcare, as some programs did in Liberia [56], or leveraging community-based day care centers or after-school programs, as was done in Malawi and South Africa [57], could help.

At the community level, the kgotla seems to be the wrong venue for information dissemination. As young women do not attend kgotla meetings, programs should investigate stakeholder recommendations for diversifying venues and means of communication to reach young women.

Many of the reported barriers were structural level barriers, including poverty, lack of gender egalitarian norms, lack of adaptation to local context, and lack of coordination. Poverty itself was a barrier to accessing and benefitting from poverty programs. The poverty level for upper middle-income countries like Botswana is set at US$165 per month [58], but poverty eradication programs generated income barely above the international threshold for *extreme* poverty set at US$57 per month (US$1.90 per day) [58]. Monthly allowances from *Ipelegeng* (US$54) and *Tirelo Sechaba* (US$60) were comparable to the extreme poverty threshold, but eligibility thresholds for programs targeting destitute people, like LIMID and the Alternative Package Program, have not been updated since the inception of the Destitute Persons Program in 1980 [59,60] and were much lower than that: US$11 (individual monthly income) and US$14 (family monthly income) [61]. It is hard to imagine that such low levels of social assistance could protect against high-risk behaviors based on economic necessity like transactional sex.

Moreover, individuals living in extreme poverty tend to spend the majority of their income on food [62]. As poverty impedes cognitive function to think through important decisions, they need consumption support before livelihood programs to have the mental bandwidth [63,64] to successfully engage in socioeconomic programs and act on HIV prevention choices. Consumption support for destitute people in Botswana is mostly offered in kind rather than in cash [61], but coupons or food baskets do not provide the flexibility to save, invest in microenterprise growth, or provide for veterinary care to safeguard flocks and stock. Combined with a lack of program monitoring, it is not surprising that participants lost their chickens or failed to benefit from microenterprise programs.

Our findings align with prevailing gender norms in many Sub-Saharan African countries that assign childcare and domestic work to women and require them to obtain male approval to engage in economic activity [65]. Other studies report similar findings and labelled Botswana a patriarchal society [66,67], although urban youth might hold more gender-equal views [68]. As patriarchal gender norms are an underlying factor for intimate partner violence, which is associated with a three-fold increase in HIV infection [69], it is essential to address the gender dimensions of promotive social protection programs. To generate male support for female economic activity, dialogue groups proposed engaging boyfriends in disseminating program information. More transformational interventions with men and boys might, however, be necessary to challenge deeply entrenched patriarchal beliefs and behaviors. In Uganda, a single day gender workshop for couples increased male support for young women’s businesses and improved male involvement in traditionally female household chores [70]. In Côte d’Ivoire, gender dialogues added to a microfinance program may have reduced intimate partner violence [71].

Young women were unlikely to apply to agricultural programs due to gender norms and a lack of interest and resources. Suggestions made in dialogue groups regarding programs leveraging positive role models or setting performance indicators for program officers to bring young women onto programs could also be used to attract young women to agriculture. Land grants, training, and linking with commercial retailers supported female farming in South Africa, where farming is strongly gendered with women traditionally undertaking most food farming whilst excluded from owning and controlling land [72]. In Malawi, farmer field schools improved farming practices through credit, savings, and training. The intervention contributed to improved food security, economic resilience, and HIV prevention among the mainly female participants [73]. Although the mean age of women in these interventions was higher than in our study, some elements might be transferable to the Botswana context. Mixed livelihood strategies might be another option. A recent study in Kenya showed that, when not forced into a binary choice of farming or other livelihood options, most rural youth saw farming play some role in their future [74].

Officers were critical of the suitability and sustainability of socioeconomic programs. To transform microenterprises into sustainable, income-earning ventures, programs need to respond to local demand and conditions [56], leverage local resources, develop markets for products, and forge private sector links [75]. If these elements would be incorporated in economic empowerment programs, young women in Botswana might benefit more from such programs. For example, assisted by local business owners, adolescent girls in Uganda developed income-generating activities adapted to local markets and increased self-employment by 72% and tripled earnings compared to the baseline [76].

Officers highlighted a lack of coordination between Botswana’s many social protection programs and with supporting services like land and water boards. These findings align with the 2013 World Bank Report on social protection in Botswana [61] and more recent studies that call for improved coordination between programs to reduce overlap, duplication and fragmentation of social protection programs [52,77]. A more holistic and client-centered approach might lead to better outcomes and more effective and efficient use of resources [78]. This would require a national strategy for HIV-sensitive social protection and an intersectoral approach to planning, implementation and co-financing of programs [79].

Although not designed for HIV prevention, programs would need to accommodate or even preferentially attract vulnerable young women to become HIV-sensitive. A holistic approach to HIV prevention and socioeconomic empowerment that places unemployed and out-of-school young women at the center may help guide them through personal, interpersonal, and structural challenges. To our knowledge, this is the first study to take an HIV-sensitive perspective on social protection in Botswana. Findings of this study will feed into a Policy Delphi of stakeholder-generated and validated proposals to improve the HIV-sensitivity of available promotive social protection programs.

### 4.1. Strengths & limitations

We triangulated methods and data sources in five different districts exploring demand and supply side perspectives [80]. Our participatory approach to identifying problems and solutions generated context-sensitive and locally feasible options to improve the situation.

Our sampling strategy may have introduced selection bias, as some young women may not have wanted to participate because they were too shy, occupied with other responsibilities, or did not receive permission from boyfriends to participate. Our interviews with a small number of young women and program officers covered only one of five implementation districts. The interviews focused on personal experience and could be subject to social desirability bias. FCM engaged stakeholders in all five districts and focused on perceptions of causes beyond personal experience. Bolstered by their peers, young women in mapping groups might have been more willing to express negative views of program officers. Although facilitators encouraged all participants to contribute, power differentials might have influenced deliberative dialogues that combined young women and officers. Policies and programs may have changed since we collected data in 2017. For example, the last BOCODOL students graduated in November 2018, after which BOCODOL changed to Botswana Open University [81].

## 5. Conclusion

Botswana’s mature and well-funded social protection system could be adapted to become more HIV-sensitive. If unemployed and out-of-school young women, who bear the brunt of HIV infections, could benefit from available promotive social protection programs that could empower them socially and economically, they might be better placed to act on HIV prevention measures. Existing programs, however, would need to become more responsive to the multiple intersecting barriers young women face at personal, interpersonal, community and structural levels that currently prevent them from benefitting from these programs. Involving vulnerable young women in program revisions could help to tailor programs to their needs.

## Supporting information

S1 Appendix FCM coding tree: Why young women do not benefit from programs. (DOCX)

S2 Appendix Deliberative dialogue: Pattern matching tables, selection & improvement recommendations. (DOCX)

S3 Appendix Framework analysis interviews. (XLSX)

S4 Appendix Script for informed oral consent for interviews and workshops (FCM and deliberative dialogue workshops). (DOCX)
