# Supplementary material for: HIV-sensitive social protection for unemployed and out-of-school young women in Botswana: An exploratory study of barriers and solutions
Source: PLoS One. 2024 Jan 10;19(1):e0293824. doi: 10.1371/journal.pone.0293824 (PMC10781194; doi:10.1371/journal.pone.0293824)
Supplement: S1 Appendix — (DOCX) [file pone.0293824.s001.docx]

**Appendix 1 FCM coding tree: why young women do not benefit from programs**

| **FCM Concepts (n=168) from 11 maps** |  |  |  | **Participant analysis** | **Researcher analysis** |
| --- | --- | --- | --- | --- | --- |
| **Concepts on both stakeholder maps mentioned at least once across districts (n=115)** | **Concepts on both stakeholder maps in at least three districts (n=37)** | **Concepts on young women maps only (n=11)** | **Concepts on service provider maps only (n=5)** | **FCM Themes**  **(n=25)** | **Overall Categories**  **(n=5)** |
| Forms in English; application process is time consuming; info unclear; difficulty completing forms; eligibility criteria (certifications); finding quotations); YW do not have information needed on form. | Complicated forms; lack of understanding forms |  |  | Application process complicated | **Program factors**   - Access to program (information) - Process issues - Programs unsuitable |
| Programs are overwhelmed; land authorities take long to allocate land. | Process is too long; assessments are too slow; no feedback; feedback takes too long; long waiting lists; when accepted it takes a long time for funds/materials to come |  |  | Application process is too long |  |
| Unsuitable projects; the grants are insufficient to make successful projects; funds insufficient for development (to pay lease, rental, debts); financial requirements of programs; few youth projects survive; no market for products; programs not specifically targeting YW |  |  | Negative effect of *Ipelegeng*: too much involvement in *Ipelegeng* means YW don’t apply for other programmes | Unsuitable projects |  |
| Program Officers (PO) wait a long time to receive funding; unspecified budgets; lack of equipment in government offices. |  |  | There is insufficient monitoring of YW’s projects | Offices lack resources |  |
| Poor program information dissemination; lack of timely program information | Program information goes to the wrong place (Kgotla, *Pitso*, Facebook); YW don’t attend Kgotla/meetings, sports & arts groups arranged by programs |  |  | Poor program information dissemination |  |
| Program offices are far away |  | POs don’t visit remote areas;  lack of transport from remote areas |  | Difficulty with physical access |  |
| **Concepts on both stakeholder maps mentioned at least once across districts** | **Concepts on both stakeholder maps in at least three districts** | **Concepts on young women maps only** | **Concepts on service provider maps only** | **FCM Themes** | **Overall Categories** |
| Improper attitudes PO; corruption; bribes; demand for sexual favours; POs are unreliable; repeated visits; |  | POs are absent |  | Bad PO behavior | **PO factors**   - Bad PO behavior - POs not helpful/ friendly - POs have negative views of YW |
| Programs look at what your family has; POs throw away forms; POs reject applications; YW applications are not successful; lack of monitoring. |  | Unfair assessment (discrimination against poor/small communities; favouritism; tribalism) |  | PO not fair |  |
| POs are unhelpful, POs don’t assist, provide, or fill out forms; PO’s ignore people; POs are rude; POs redirect you to other education; local offices set earlier deadlines; POs bad time management; stress; lack of training |  |  |  | PO not helpful |  |
| POs hate YW; POs think YW are irresponsible; POs think YW are not serious |  |  |  | PO negative attitudes about YW |  |
| Lack of cooperation between POs and YW; POs are too old |  | Language barriers between PO & YW |  | Lack of cooperation YW-PO |  |
| YW do not know where to get information | Lack of knowledge about programs |  |  | YW lack of knowledge | **YW factors**   - Lack of knowledge and skills - Lack of self-confidence - Unhelpful attitudes and behaviors YW - YW have negative views of programs |
| YW have poor literacy; lack experience; lack entrepreneurial skills; no skills in writing business plan; no skills looking after livestock; lack of communication skills | Lack of skills; YW lack (formal) education |  |  | YW lack skills required for programs |  |
| YW lack courage, assertiveness; fear visiting program offices; fear projects are too big for them; fear being unable to repay loans; lack of communication skills | YW lack confidence; lack self-esteem; are shy; fear of rejection; fear failure of the project | YW don’t ask questions | Trauma (painful experience (rape) makes them hesitant) | YW lack confidence |  |
| YW give up, undermine programs, don’t look after their projects; YW prefer having a good time; YW need fast cash; few youth projects survive; youth don’t look after projects | YW lack commitment | YW are indecisive /fail to prioritize;  YW move around a lot |  | YW lack commitment |  |
| YW have wrong perceptions about programs; YW find programs too time consuming / demanding; YW don’t think they can choose; YW are not into arable farming; YW don’t want to do dirty jobs (agriculture); YW prefer white collar jobs |  | Program names are a ‘turn off’- poverty eradication and LIMID make you look poor; programs are stigmatizing |  | YW have negative attitudes about programs |  |
| YW are not proactive; lack business passion; YW want quick money; need fast cash; YW are too impatient to wait for feedback; YW procrastinate; YW are disrespectful; YW have bad attitudes; YW use (excessive) alcohol and drugs. | YW are not interested; YW are impatient; YW are lazy |  |  | YW unhelpful attitudes/behavior |  |
| **Concepts on both stakeholder maps mentioned at least once across districts** | **Concepts on both stakeholder maps in at least three districts** | **Concepts on young women maps only** | **Concepts on service provider maps only** | **FCM Themes** | **Overall Categories** |
| YW tell others of bad experiences; YW see others being rejected; YW hear that it takes long to receive money-we then sit down; peer pressure (to not apply); theft of stock and materials; security concerns for projects in the bush/remote areas |  | Program names are a ‘turn off’- poverty eradication and LIMID make you look poor; programs are stigmatizing |  | Stories of negative experiences | **Social factors**   - Competing household/ childcare responsibilities - Problems with partners - Negative peer influence (stories & stigma) - Jealousy & competition between YW |
| YW do not collaborate; YW are unwilling to share information; YW do not support peers |  | YW compete for clients;  YW are jealous |  | Competition between YW |  |
| YW lack family support | Household responsibilities YW; juggling responsibilities; YW look after young children; YW have problems with childcare; childbearing/pregnancy YW |  |  | Household and child responsibilities YW |  |
| YW are too dependent on partners; YW have difficult partners; boyfriends stop us; boyfriend refused to give me permission to attend a meeting |  |  |  | Problems with partners |  |
| **Concepts on both stakeholder maps mentioned at least once across districts** | **Concepts on both stakeholder maps in at least three districts** | **Concepts on young women maps only** | **Concepts on service provider maps only** | **FCM Themes** | **Overall Categories** |
| YW need start-up funding; financial requirements programs exclude YW; some projects are very expensive; YW have no equipment | Lack of money; YW have no funds to start project; YW experience more expenses when applying; YW have no money for transportation to organize quotations |  |  | Lack of resources to apply | **Structural factors**   - YW lack resources to apply (poverty) - Gender roles YW (social norms) - Institutional barriers like: - Programs lack resources like land - Lack of coordination |
| Societal norms; cultural beliefs; don’t expect YW to do such projects; YW lack community support; agriculture is traditionally for men; YW don’t look after cattle; YW don’t think livestock programs are for them; arable farming is for old people; household responsibilities of young women; YW have no enabling environment; religious inclinations; YW lack role models |  |  |  | Gender roles YW |  |
| YW do not have access to water; YW are not applying for land; environmental conditions unsuitable (farming); YW don’t have or don’t apply for land | YW do not have access to (ploughing) land |  | Lack of political will/ strict laws (excluding YW) | Lack resources for programs |  |
|  |  |  | Lack of coordination (between programs; poor supply-side coordination; poor communications between programs & departments);  Policies and legislation issues (excluding YW) | Lack of coordination |  |
