# Supplementary material for: HIV-sensitive social protection for unemployed and out-of-school young women in Botswana: An exploratory study of barriers and solutions
Source: PLoS One. 2024 Jan 10;19(1):e0293824. doi: 10.1371/journal.pone.0293824 (PMC10781194; doi:10.1371/journal.pone.0293824)
Supplement: S2 Appendix — (DOCX) [file pone.0293824.s002.docx]

**Appendix 2. Deliberative dialogue: pattern matching tables, selection & improvement recommentdations**

**Pattern matching tables: concepts mentioned by young women**

|  | District 1 | | District 2 | District 3 | District 4 | District 5 |
| --- | --- | --- | --- | --- | --- | --- |
| Program Factors | YW1 | YW2 |  |  |  |  |
| Process & forms are complicated |  |  | ✓ | ✓ |  | ✓ |
| Complicated application processes: application is time consuming |  |  | ✓ |  |  | ✓ |
| Complicated forms; difficult forms; in English |  |  | ✓ | ✓ |  | ✓ |
| Process & forms are complicated |  |  |  |  |  |  |
| Lack of understanding of forms |  |  |  | ✓ |  |  |
| Long delays | ✓ | ✓ | ✓ | ✓ |  | ✓ |
| Process is too long; time consuming |  |  |  | ✓ |  | ✓ |
| Long delays: in approval, in funding coming through | ✓ | ✓ | ✓ |  |  | ✓ |
| No feedback on applications | ✓ | ✓ |  |  |  |  |
| Lack of communication from programs |  |  |  |  |  | ✓ |
| No suitable projects available | ✓ | ✓ |  |  |  |  |
| Unsuitable projects |  | ✓ |  |  |  |  |
| Amounts allowed (e.g., for goats) are not enough – quotes are higher |  | ✓ |  |  |  |  |
| Poor information dissemination | ✓ | ✓ | ✓ | ✓ | ✓ | ✓ |
| YW lack knowledge about programmes/reaching YW; lack of media/poor information dissemination | ✓ | ✓ | ✓ | ✓ |  | ✓ |
| Information about programs not clear |  |  | ✓ |  | ✓ |  |
| Methods of communication don’t reach YW – they don’t attend kgotla, listen to RB1, watch BTV or have Facebook access | ✓ | ✓ |  |  |  |  |
| Local offices set earlier deadlines than the national deadlines |  |  |  |  | ✓ |  |
| Difficulty physical access (remote areas) | ✓ | ✓ | ✓ | ✓ | ✓ | ✓ |
| POs don’t visit remote areas |  |  | ✓ |  | ✓ |  |
| The programme offices are far away; YW stay far from services | ✓ | ✓ | ✓ | ✓ | ✓ | ✓ |
|  | District 1 | | District 2 | District 3 | District 4 | District 5 |
| Program officer factors | YW1 | YW2 |  |  |  |  |
| PO bad behaviour | ✓ |  | ✓ | ✓ | ✓ |  |
| Programme officers demand sexual favours; improper attitudes; bad PO behavior; POs demand bribes | ✓ |  | ✓ | ✓ | ✓ |  |
| PO not helpful/client friendly | ✓ | ✓ | ✓ | ✓ | ✓ | ✓ |
| Program officers not helpful; bad attitudes; poor reception | ✓ | ✓ | ✓ | ✓ | ✓ | ✓ |
| No assistance to complete the forms |  |  |  | ✓ |  |  |
| POs lack communication skills |  |  | ✓ |  | ✓ |  |
| Time management of POs not good |  |  |  |  |  | ✓ |
| PO have negative views of YW |  |  | ✓ |  |  |  |
| POs think YW are irresponsible/have negative attitudes about YW |  |  | ✓ |  |  |  |
| PO absent |  |  |  | ✓ |  |  |
| POs not in office, especially on Fridays |  |  |  | ✓ |  |  |
| PO unfair | ✓ | ✓ | ✓ |  | ✓ | ✓ |
| Assessment of applications is not fair | ✓ | ✓ |  |  |  |  |
| POs favour some people; tribalism; discrimination of the poor |  |  | ✓ |  |  | ✓ |
| Programme officers “throw away applications forms” | ✓ | ✓ |  |  |  |  |
| Eligibility decisions not fair – they reject our applications |  |  |  |  |  | ✓ |
| Applications of YW are not successful |  |  |  |  | ✓ |  |
| Lack cooperation |  |  | ✓ | ✓ |  |  |
| Lack of cooperation between YW and POs |  |  |  | ✓ |  |  |
| Language barrier (e.g., *Setswana, Sekalanga*) |  |  | ✓ | ✓ |  |  |
| PO personal situation |  |  |  |  |  | ✓ |
| Stress of POs |  |  |  |  |  | ✓ |
|  | District 1 | | District 2 | District 3 | District 4 | District 5 |
| Young women factors | YW1 | YW2 |  |  |  |  |
| Lack of knowledge and skills |  |  | ✓ | ✓ | ✓ | ✓ |
| Lack of formal education of YW |  |  | ✓ |  |  |  |
| Poor literacy of young women |  |  |  | ✓ |  |  |
| YW lack required skills for projects |  |  |  | ✓ |  | ✓ |
| YW lack communication skills |  |  | ✓ | ✓ |  |  |
| YW lack of confidence | ✓ | ✓ | ✓ | ✓ | ✓ | ✓ |
| Fear of failure of projects/rejection/confidence |  |  | ✓ | ✓ |  |  |
| YW have low self-esteem; shy | ✓ | ✓ |  | ✓ | ✓ | ✓ |
| Difficult to ask questions/ YW don’t ask questions |  |  |  |  | ✓ | ✓ |
| YW don’t apply / reluctant to apply – negative perceptions |  |  |  |  | ✓ |  |
| Theft of stock – worry can’t repay loan |  |  |  |  |  | ✓ |
| YW Lack of commitment | ✓ | ✓ | ✓ | ✓ |  | ✓ |
| Young women are lazy, lack interest and commitment | ✓ | ✓ | ✓ | ✓ |  | ✓ |
| Some YW mismanage projects |  |  | ✓ |  |  |  |
| YW can’t make choices |  |  |  | ✓ |  |  |
| YW have negative perceptions/attitudes about the program |  |  | ✓ | ✓ |  | ✓ |
| Wrong perceptions of programmes |  |  |  | ✓ |  |  |
| Negative attitude of YW – stigma of some programs – seen as dirty jobs |  |  |  |  |  | ✓ |
| YW ashamed to be seen on some programmes (eg LIMID, Poverty Eradication) |  |  |  | ✓ |  |  |
| YW think programs are too demanding |  |  | ✓ |  |  |  |
| Lack of cooperation between YW and POs |  |  |  | ✓ |  |  |
| YW unhelpful attitudes and behaviour |  |  | ✓ | ✓ |  | ✓ |
| Young women are not respectful |  |  | ✓ |  |  |  |
| YW lack patience |  |  |  | ✓ |  | ✓ |
| Young women move around a lot |  |  | ✓ |  |  |  |
| YW like partying, drugs and alcohol |  |  | ✓ |  |  |  |
| YW don’t prioritise these programs – rely on what their family has |  |  |  |  |  | ✓ |
|  |  |  |  |  |  |  |
|  | District 1 | | District 2 | District 3 | District 4 | District 5 |
| Social factors | YW1 | YW2 |  |  |  |  |
| Household and child responsibilities | ✓ | ✓ | ✓ | ✓ |  | ✓ |
| YW have no support from family/parental support | ✓ | ✓ | ✓ | ✓ |  | ✓ |
| Looking after young children – lack of childcare arrangements; teenage pregnancy; young age pregnancy |  |  | ✓ | ✓ |  | ✓ |
| Lack of community support | ✓ | ✓ | ✓ |  |  |  |
| YW don’t get support from leaders/community; social support | ✓ | ✓ | ✓ |  |  |  |
| Problems with partners | ✓ |  | ✓ | ✓ |  | ✓ |
| YW rely too much on boyfriends/men |  |  |  |  |  | ✓ |
| Boyfriends don’t allow YW to access programmes; too dependent on partners; difficult partners | ✓ |  | ✓ | ✓ |  |  |
| Negative peer influence | ✓ | ✓ |  | ✓ | ✓ | ✓ |
| YW talk down the programmes to their peers; Peer pressure (not to apply) | ✓ |  |  | ✓ |  |  |
| see others rejected |  |  |  |  | ✓ |  |
| Alcohol and drugs use by YW |  |  |  | ✓ |  | ✓ |
| Peer pressure - stigma |  |  |  |  |  | ✓ |
| Other YW are jealous (they refuse to share information with others); YW don’t cooperate with each other |  | ✓ |  |  |  | ✓ |
| Projects fail because of competition for clients/markets |  | ✓ |  | ✓ |  |  |
|  | District 1 | | District 2 | District 3 | District 4 | District 5 |
| Structural factors | YW1 | YW2 |  |  |  |  |
| Poverty | ✓ | ✓ | ✓ | ✓ |  | ✓ |
| YW lack money for the expenses associated with the applications | ✓ | ✓ | ✓ |  |  | ✓ |
| Insufficient funds for the programs |  |  | ✓ |  |  |  |
| Lack of funds for project expenses |  |  |  | ✓ |  |  |
| Social norms |  |  |  | ✓ |  |  |
| Gender roles /social expectations |  |  |  | ✓ |  |  |
| Institutional barriers | ✓ |  | ✓ | ✓ | ✓ | ✓ |
| No access to water (for LIMID, ISPAAD), lack of land | ✓ |  | ✓ | ✓ | ✓ | ✓ |
| Lack of equipment in offices |  |  |  |  |  | ✓ |
| Environmental barriers |  |  |  | ✓ |  | ✓ |
| Lack of transport to reach offices |  |  |  | ✓ |  |  |
| Theft of stock – worry can’t repay loan |  |  |  |  |  | ✓ |
|  |  |  |  |  |  |  |

*In Purple: concepts only mentioned by young women*

**Pattern matching tables: concepts mentioned by service providers**

|  | District 1 | District 2 | District 3 | District 4 | District 5 |
| --- | --- | --- | --- | --- | --- |
| Program Factors |  |  |  |  |  |
| Process & forms are complicated | ✓ | ✓ |  | ✓ | ✓ |
| Complicated application processes: long process– e.g., company registration causes delays; Lack of knowledge to write business proposal |  | ✓ |  | ✓ |  |
| Application forms are difficult (esp. YDF); in English; | ✓ | ✓ |  |  | ✓ |
| Long delays: in approval, in funding coming through | ✓ | ✓ |  |  | ✓ |
| Unsuitable projects | ✓ | ✓ | ✓ |  |  |
| Financial requirements of programs; Lack of funds for project expenses |  | ✓ | ✓ |  |  |
| The grants are not enough to make successful projects | ✓ |  |  |  |  |
| Negative effect of *Ipelegeng* – don’t apply for other programmes; too much *Ipelegeng* involvement | ✓ | ✓ |  |  |  |
| Poor information dissemination | ✓ | ✓ | ✓ | ✓ | ✓ |
| Lack of information about programs reaching YW; lack of media |  | ✓ | ✓ |  | ✓ |
| Young women don’t go the kgotla/*pitso* |  | ✓ | ✓ |  |  |
| Methods of communication don’t reach YW – they don’t attend kgotla, listen to RB1, watch BTV or have Facebook access | ✓ |  |  |  |  |
| YW not interested in arts or sports groups |  |  |  | ✓ |  |
| Difficulty physical access (remote areas) |  | ✓ | ✓ |  |  |
| YW stay far from services; offices are centralised |  | ✓ | ✓ |  |  |
|  | District 1 | District 2 | District 3 | District 4 | District 5 |
| Program Officer Factors |  |  |  |  |  |
| PO bad behaviour |  |  | ✓ |  |  |
| Improper attitude of POs |  |  | ✓ |  |  |
| Program officers not helpful; poor reception |  | ✓ | ✓ |  |  |
| POs think YW are irresponsible/have negative attitudes about YW |  | ✓ |  |  |  |
| Lack of cooperation between YW and POs |  |  | ✓ |  |  |
|  | District 1 | District 2 | District 3 | District 4 | District 5 |
| Young women Factors |  |  |  |  |  |
| Lack of knowledge and skills | ✓ | ✓ | ✓ | ✓ | ✓ |
| YW lack knowledge about programmes | ✓ |  | ✓ |  |  |
| YW lack of required skills | ✓ | ✓ |  | ✓ | ✓ |
| YW lack education | ✓ | ✓ |  |  |  |
| lack of (entrepreneurial) skills |  |  |  | ✓ | ✓ |
| YW lack of confidence | ✓ | ✓ | ✓ | ✓ | ✓ |
| YW have low self-esteem, shy | ✓ |  | ✓ | ✓ | ✓ |
| Fear of failure/rejection; lack of confidence |  | ✓ |  |  |  |
| Theft of stock – worry can’t repay loan |  |  |  |  | ✓ |
| YW Lack of commitment | ✓ | ✓ | ✓ | ✓ | ✓ |
| Young women are lazy, lack interest and commitment, irresponsible | ✓ | ✓ | ✓ |  | ✓ |
| Few youth projects survive |  |  |  | ✓ |  |
| Youth don’t look after projects |  |  |  | ✓ |  |
| Not enough monitoring of those on programs |  |  |  | ✓ |  |
| YW have negative perceptions/attitudes about the program | ✓ |  | ✓ | ✓ | ✓ |
| YW don’t apply / reluctant to apply /see other rejected– negative perceptions |  |  |  | ✓ |  |
| Wrong perceptions of programmes |  |  | ✓ |  |  |
| YW don’t want to do dirty jobs (e.g., ISPAAD); YW prefer white collar jobs / not into arable farming; stigma of some programs (seen as dirty jobs | ✓ |  | ✓ |  | ✓ |
| The required work for the programs is time consuming |  |  |  |  | ✓ |
| Lack of cooperation between YW and POs |  |  | ✓ |  |  |
| YW unhelpful attitudes and behaviour |  | ✓ |  | ✓ | ✓ |
| YW need fast cash |  |  |  | ✓ |  |
| YW are too impatient to wait for feedback |  |  |  | ✓ | ✓ |
| YW like partying, drugs, and alcohol |  | ✓ |  |  |  |
| YW don’t prioritise these programs – rely on what their family has |  |  |  |  | ✓ |
| Painful experiences (e.g., rape) make them hesitant |  | ✓ |  |  |  |
|  | District 1 | District 2 | District 3 | District 4 | District 5 |
| Social Factors |  |  |  |  |  |
| Household and child responsibilities | ✓ | ✓ | ✓ | ✓ | ✓ |
| YW have no support from family/parents | ✓ | ✓ | ✓ |  |  |
| Looking after young children – lack of childcare arrangements; teenage pregnancy; young age pregnancy |  | ✓ | ✓ |  | ✓ |
| Household responsibilities of young women |  |  |  | ✓ |  |
| Lack of community support | ✓ | ✓ |  |  |  |
| YW don’t get support from leaders/community; social support | ✓ | ✓ |  |  |  |
| Problems with partners |  | ✓ | ✓ |  |  |
| Boyfriends stop them applying/too dependent on partners; difficult partners |  | ✓ | ✓ |  |  |
| Negative peer influence |  |  |  | ✓ | ✓ |
| see others rejected |  |  |  | ✓ |  |
| Peer pressure – stigma |  |  |  |  | ✓ |
|  | District 1 | District 2 | District 3 | District 4 | District 5 |
| Structural Factors |  |  |  |  |  |
| Poverty | ✓ | ✓ | ✓ |  |  |
| YW lack money for the expenses associated with the applications | ✓ | ✓ |  |  |  |
| Lack of funds for project expenses |  |  | ✓ |  |  |
| Social norms | ✓ | ✓ | ✓ |  |  |
| Societal norms against women doing such projects; Cultural beliefs and norms don’t expect YW to do such projects | ✓ | ✓ | ✓ |  |  |
| Institutional barriers | ✓ | ✓ | ✓ | ✓ | ✓ |
| Lack of coordination between programmes | ✓ |  |  |  |  |
| Policies and legislation issues (make it difficult to access the programmes) | ✓ |  |  |  |  |
| Insufficient funds for the programs; |  | ✓ |  |  | ✓ |
| YW don’t have or don’t apply for land | ✓ | ✓ | ✓ | ✓ | ✓ |
| No access to water (for LIMID, ISPAAD) | ✓ |  |  |  |  |
| Environmental barriers |  | ✓ |  |  | ✓ |
| Security concerns for projects in the bush/remote areas |  | ✓ |  |  |  |
| Theft of stock – worry can’t repay loan |  |  |  |  | ✓ |
|  |  |  |  |  |  |

*In Blue: concepts only mentioned by service providers*

**Pattern matching tables: concepts mentioned by both stakeholder groups**

|  | District 1 | | | District 2 | | District 3 | | District 4 | | District 5 | |
| --- | --- | --- | --- | --- | --- | --- | --- | --- | --- | --- | --- |
|  | YW 1 | YW2 | PO | YW | PO | YW | PO | YW | PO | YW | PO |
| YW have low self-esteem, lack of confidence, shy, fear of failure/rejection, worry to be unable to repay loans | ✓ | ✓ | ✓ | ✓ | ✓ | ✓ | ✓ | ✓ | ✓ | ✓ | ✓ |
| YW lack required resources (land, water), don’t apply to land | ✓ |  | ✓ | ✓ | ✓ | ✓ | ✓ | ✓ | ✓ | ✓ | ✓ |
| (Officers think that) YW are lazy, irresponsible, impatient, lack interest and commitment, want fast cash | ✓ | ✓ | ✓ | ✓ | ✓ | ✓ | ✓ |  |  | ✓ | ✓ |
| Long delays: in approval, in funding coming through | ✓ | ✓ | ✓ | ✓ | ✓ |  |  | ✓ | ✓ | ✓ | ✓ |
| Lack of information. Program information doesn’t reach YW – they don’t attend kgotla/listen to Radio Botswana 1 / watch Botswana TV / have Facebook access | ✓ | ✓ | ✓ | ✓ | ✓ | ✓ | ✓ |  |  | ✓ | ✓ |
| Cultural beliefs, social norms, YW don’t do such projects | ✓ | ✓ | ✓ | ✓ | ✓ | ✓ | ✓ |  |  | ✓ | ✓ |
| YW have no support from family (household, childcare tasks) | ✓ | ✓ | ✓ | ✓ | ✓ | ✓ | ✓ |  |  |  |  |
| YW lack knowledge / skills | ✓ | ✓ | ✓ |  |  |  |  | ✓ | ✓ | ✓ | ✓ |
| Application forms are difficult (YDF, in English) | ✓ | ✓ | ✓ | ✓ | ✓ |  |  |  |  | ✓ | ✓ |
| YW lack money for the expenses associated with applications | ✓ | ✓ | ✓ | ✓ | ✓ |  |  |  |  | ✓ | ✓ |
| YW stay far away from services |  |  |  | ✓ | ✓ | ✓ | ✓ |  |  |  |  |
| Program officers not helpful, poor reception, improper attitudes |  |  |  | ✓ | ✓ | ✓ | ✓ |  |  |  |  |
| YW have wrong/negative perception of programs |  |  |  |  |  | ✓ | ✓ |  |  | ✓ | ✓ |
| YW like partying, drugs, and alcohol; drug/alcohol abuse |  |  |  | ✓ | ✓ |  |  |  |  | ✓ | ✓ |
| Boyfriends stop them from applying |  |  |  | ✓ | ✓ | ✓ | ✓ |  |  |  |  |
| YW don’t look after cattle, no dirty jobs, want white collar jobs |  |  |  |  |  | ✓ | ✓ |  |  | ✓ | ✓ |
| YW do not get support from leaders/community | ✓ | ✓ | ✓ |  |  |  |  |  |  |  |  |
| Financial requirements of programs (own capital) |  |  |  | ✓ | ✓ |  |  |  |  |  |  |
| YW lack formal education |  |  |  | ✓ | ✓ |  |  |  |  |  |  |
| YW lack funds for project expenses |  |  |  |  |  | ✓ | ✓ |  |  |  |  |
| Lack of cooperation between YW and officers |  |  |  |  |  | ✓ | ✓ |  |  |  |  |
| YW don’t apply -see others rejected |  |  |  |  |  |  |  | ✓ | ✓ |  |  |
| Peer pressure, stigma |  |  |  |  |  |  |  |  |  | ✓ | ✓ |
| Teenage pregnancy -problems with childcare |  |  |  |  |  |  |  |  |  | ✓ | ✓ |
| YW don’t prioritize these programs -rely on what family has |  |  |  |  |  |  |  |  |  | ✓ | ✓ |
|  |  |  |  |  |  |  |  |  |  |  |  |

Deliberative dialogue workshops listed issues mentioned by both stakeholder groups in a given INSTRUCT intervention district. From this list participants chose two to four issues to discuss potential solutions. YW: unemployed and out-of-school young women.

Concepts mentioned by *both* groups in red: 5/5 districts; blue: 4/5 districts; green: 3/5 districts; orange: 2/5 districts; **black**: 1/5 districts

**Issues selected for deliberative dialogue and improvement recommendations**

|  | **District 1** | **District 2** | **District 3** | **District 4** | **District 5** |
| --- | --- | --- | --- | --- | --- |
| 1 | Lack of outreach | Lack of outreach | Lack of outreach | Lack of outreach | Lack of outreach |
| 2 |  | Difficult forms | Difficult forms | Difficult forms | *Difficult forms (not discussed)* |
| 3 |  |  |  |  | Lack of skills -need for training |
| 4 | Lack of education |  |  |  |  |
| 5 |  | Unhelpful PO |  |  |  |
| 6 | Lack of Land |  |  |  |  |
| 7 |  | *Low self-esteem YW (not discussed)* |  |  |  |
|  | **1. Improve outreach**  Preference for outreach at VDC but programs are obliged to go to Kgotla: possibility of 2 visits: Kgotla/ other venue.  Other means to get out information   - Use a megaphone - Youth form support groups and PO could come and visit them - Use SMS – sending out messages that go to everyone   **4. Lack of education**  Need to make YW aware that they don’t have to pay for BOCODOL.  If problem with completing forms is literacy, go to OSET to learn to read and write, or ask someone for help.  **5. Lack of land**   - Programs and sub-land board could collaborate (formal agreements) - Programs responsible for the lease - District commissioner could coordinate– people would then attend – officers to report progress on applications to each program and access to land - YW could form groups, collect information about communal land needs and bring this information to program officers who could bring it to district coordination meetings. - Land board could assign appropriate land for 1-4 YW (already developed) - Needs action at national level as well | **1. Improve outreach**  “Taking service to the people” events:  In the community hall or in schools  Flyers/posters. Liaise with councillors. Tell kids in school so they can pass the word.  Organize a football tournament and ask young men to bring YW with them  Social media: make a Facebook page in the district as a one-stop shop with all programs posting information and updates. YW could form groups around the Facebook page in each community. YDF could fund such groups.  **2. Assistance to complete forms**  The form has been improved already: it is now in both Setswana and English and reduced to 11 rather than 22 pages.  The YDF form is still very difficult and YW need someone to help them complete it.  **5. Unhelpful program officers**   - Improve turnaround time - Improve record keeping - Tackle corruption/serious inefficiency - Improve the reception given to YW | **1. Improve outreach**  Programs to undertake outreach to all communities and settlements –to work together  Community mobilization for outreach sessions: VDCs, Kgosi, youth committee, VHC, churches.  Groups of YW in each community to call program officers to come for training (Ipiletso).  Nurses to talk to YW when they do mobile stops in the *masimo* (arable lands outside village)*.*  School kids to pass on messages.  Involve boyfriends as well as YW  Organize market days to display successful projects in all communities  Social media: access is limited to towns and large villages. YW who have access could help those who don’t have access to Facebook.  All programs in the district in one place & regular updates.  Measure program officers’ objective to bring YW onto programs  **2. Assistance to complete forms**  Program officers could train two YW per community about the programs and about how to complete the forms (training of trainers)  Then these YW can train their peers about the programs and about how to complete forms. | **1. Improve outreach**  Health promotion officer (DHMT) to work with 2 YW to collect information from all programs in the district, collate it and use clinics to reach YW in the community, as YW attend child welfare there.  Also use notice boards at the Kgotla, post office, community hall, and police station.  **2. Assistance to complete forms**  Train two YW to complete YDF forms. These two can assist other YW in the community completing forms. | **1. Improve outreach**  YW can elect representatives to be in the Kgotla to collate program information and prepare it for dissemination by small groups of YW in Dikgotlana groups (small Kgotla spread out around the community) to pass it on to girls in smaller communities.  **3. Lack of skills in YW**  YW need to group themselves and approach program officers for training. LIMID can provide training in animal husbandry. ISPAAD has different training programs on offer like leather bag making (6-m program), yoghurt manufacturing (1 day), and breeding goats. |

YW: unemployed and out-of-school young women; VDC: village development committees; VHC: village health committee; DHMT: district health management team.
