# Supplementary material for: HIV-sensitive social protection for unemployed and out-of-school young women in Botswana: An exploratory study of barriers and solutions
Source: PLoS One. 2024 Jan 10;19(1):e0293824. doi: 10.1371/journal.pone.0293824 (PMC10781194; doi:10.1371/journal.pone.0293824)
Supplement: S4 Appendix — (DOCX) [file pone.0293824.s004.docx]

**Appendix 4: Script for informed oral consent for interviews and workshops (FCM & deliberative dialogue workshops)**

**Prepared script to be read by interviewers prior to participation of participant.**

*Interviews with unemployed and out-of-school young women*

**Introduction**

Hello, my name is ………... and I am working with CIET. We are working on a project with NACA to try to help young women get more access to government support programmes (like ISPAAD, LIMID, TS, getting back to school etc). I would like to ask you about your experience with applying to such government programmes. Your views and experiences of the programmes will help to look for ways to make these programmes more accessible for young women like you. Everything you say is confidential and I won’t write your name with your answers.

**Informed consent**

- Do you understand the purpose of this interview?
- Do you understand that your answers are confidential, that you don’t have to answer any question you don’t want to, and that you can stop this interview any time?
- Do you agree to participate?

*Interviews with program officers*

**Introduction**

Hello, my name is …………. and I am working with CIET. We are working on a project with NACA to try to help young women get more access to government support programmes.

I would like to ask you about your experiences as an officer in one of the government support programmes. We are interested in your views, as someone expert in how these programmes work on the ground, about what is working well in the programmes and what is not working well. All your responses are completely confidential, and we will not identify you with any comments you might make during this interview.

**Informed consent**

- Do you understand the purpose of this interview?
- Do you understand that your answers are confidential, that you don’t have to answer any question you don’t want to, and that you can stop this interview any time?
- Do you agree to participate?

**Facilitator script for oral consent - FCM**

*Script for young women who participate in fuzzy cognitive mapping (FCM) workshops*

Please use this script to explain to each potential workshop participant the purpose of the workshop and their rights as a participant, and to seek their informed consent to participate in the workshop.

We would like to invite you to participate in a FCM workshop carried out by CIET (a research group), working with NACA (the National AIDS Coordinating Agency). We are working with young women (18 to 29 years) to help them to make use of social support programs.

FCM is a group activity with about 5 to 10 people similar to yourself, in which you will share with the group what you think are reasons why young women do not benefit from social support programs. We are also interested to know what you think would help young women to benefit from these programs. You will write down your ideas on a card. Afterwards, you will compare your cards with the cards of other workshop participants. Together with other participants you will group the cards with similar ideas. You will then stick these cards on a board and draw arrows to show how the ideas on these cards link to the main idea of young women not benefitting from social support programs. As a group, you will discuss how important these links are by placing a number next to the lines with arrows. You can choose from number 1 (not very important) to number 5 (extremely important).

We will not audio record the discussion in the group. We will take notes of what is said, but without identifying who said what. At the end, we will take pictures of the maps.

At the beginning of the group discussion, we will ask everyone to respect the confidentiality of the discussion and to not repeat to people outside the group what someone shared with the group during the group discussion.

Your participation in the group is completely voluntary. If you join the group, you can leave at any time you wish to. We plan to hold the group in [location in the community] at [time planned for the group to start]. The mapping activity will take about two hours.

There is no financial compensation for your participation in the workshop, but we will provide lunch and tea. We will collect you from home and bring you back home after the workshop.

If you have any questions, you can reach us at [phone number of PI or project coordinator].

• Do you understand the purpose of the group activity and discussion? (Y/N)

• Do you understand that we will treat the discussion as confidential, and that you can leave the group at any point? (Y/N)

• Do you agree to participate in the group activity and discussion? (Y/N)

**Facilitator script for oral consent - deliberative dialogue**

*Script for all participants who participate in deliberative dialogue workshops*

Please use this script to explain to each potential workshop participant the purpose of the workshop and their rights as a participant, and to seek their informed consent to participate in the workshop.

We would like to invite you to participate in a deliberative dialogue workshop carried out by CIET (a research group), working with NACA (the National AIDS Coordinating Agency). We are working with young women (18 to 29 years) to help them to make use of social support programs.

Deliberative dialogue is a group activity with about 10 to 15 people, which includes a mix of young women and program officers. In deliberative dialogue workshops you will help analyze FCMs. We ask you to compare the maps that were made by young women with maps that program officers made. The CIET facilitator will first ask you as a group to make a list with ideas that are similar in all maps. Then you will make a list of ideas that are different between maps. As a group you will discuss these similarities and differences and select the most important issues you would like to talk about as a group.

For each of the important issues you selected you will think of how this issue could be solved. The CIET facilitator will give each participant the opportunity to share her or his views. You can say that you have nothing to say about some issues, but we would like you to share your ideas as much as you can. Sometimes you will agree with suggestions other workshop participants make, but sometimes you will not. It is important for us to know that you do not agree with what is said and why, because you may know things other people do not. It is also important that you are respectful towards other workshop participants and treat them the way you would like to be treated yourself.

Once you listed potential solutions, you will discuss as a group how you could apply those solutions in your community. If you think of solutions you can apply only with assistance of the government, CIET and NACA could discuss these solutions with the government in Gaborone.

We will not audio record the discussion in the group. We will take notes of what is said, but without identifying who said what. At the end, we will take pictures of the lists you made.

At the beginning of the group discussion, we will ask everyone to respect the confidentiality of the discussion.

Your participation in the group is completely voluntary. If you join the group, you can leave at any time you wish to. We plan to hold the group in [location in the community] at [time planned for the group to start]. The deliberative dialogue activity will take about three hours.

There is no financial compensation for your participation in the workshop, but we will provide lunch and tea. We will collect you from home and bring you back home after the workshop.

If you have any questions, you can reach us at [phone number of PI or project coordinator].

• Do you understand the purpose of the group activity and discussion? (Y/N)

• Do you understand that we will treat the discussion as confidential, and that you can leave the group at any point? (Y/N)

• Do you agree to participate in the group activity and discussion? (Y/N)
